# Case report: Acquired resistance to crizotinib from a MET Y1230H mutation in a patient with non-small cell lung cancer and *KIF5B-MET* fusion

**DOI:** 10.3389/fonc.2024.1370901

**Published:** 2024-04-16

**Authors:** Su-Su Dong, Wen Dong, Ya-Fen Tan, Qiang Xiao, Tian-Li Wang

**Affiliations:** ^1^Department of Respiratory Medicine, Changde Hospital, Xiangya School of Medicine, Central South University (the First People’s Hospital of Changde City), Changde, Hunan, China; ^2^Department of Oncology, Changde Hospital, Xiangya School of Medicine, Central South University (the First People’s Hospital of Changde City), Changde, Hunan, China

**Keywords:** acquired resistance, MET fusion, non-small cell lung cancer, gene mutations, crizotinib

## Abstract

**Background:**

The c-met proto-oncogene (*MET*) serves as a significant primary oncogenic driver in non-small cell lung cancer (NSCLC) and has the potential to fuse with other genes, such as *KIF5B*, although it occurs infrequently. Only a limited number of reported cases have examined the clinical efficacy of crizotinib in patients with *KIF5B-MET* gene fusion, with no known data regarding acquired resistance to crizotinib and its potential mechanisms. In this report, we present the clinical progression of a female patient diagnosed with NSCLC and harboring a *KIF5B-MET* gene fusion.

**Case description:**

The patient initially exhibited partial response to first-line crizotinib treatment, albeit for a short duration and with limited efficacy. Subsequent disease progression revealed the emergence of a secondary *MET* mutation, specifically MET Y1230H, leading to acquired resistance to crizotinib.

**Conclusion:**

The reporting of this case is imperative for informing clinical practice, given the uncommon occurrence of NSCLC with *MET* fusion, displaying responsiveness to MET tyrosine kinase inhibitor therapy, as well as the emergence of the secondary Y1230H alteration as a potential resistance mechanism.

## 1 Introduction

*MET* is an important primary oncogenic driver gene in non-small cell lung cancer (NSCLC). The most common *MET* aberrations are gene amplifications and exon 14 splice variants. *MET* fusion is a rare type of structural rearrangement; nine *MET* fusion partner genes have been identified, namely: *PRKAR2B* (1), *KIF5B* (2–4), *STARD3NL* (5), *CDR2* (6), *UBE2H* (7), *HLA-DRB1* (8), *ATXN7L1* (9), *CD47* (10), and *SPECC1L* (11). To date, eight cases of *KIF5B*-MET gene fusion have been reported in the literature, of which only a few have described the efficacy of crizotinib (**Table 1**). Continued reporting of these cases is necessary to inform clinical practice. Herein, we present the case of a patient who presented with neck pain and limb myasthenia persisting for more than 6 months. She was diagnosed with advanced lung adenocarcinoma with *KIF5B*-MET fusion and achieved partial response to crizotinib for 4 months. In addition, we identified a secondary *MET* mutation (MET Y1230H) after disease progression during crizotinib therapy. To the best of our knowledge, this is the first clinical report of a MET Y1230H mutation arising in a patient with *KIF5B-MET* fusion.

**Table 1:** Characteristics of patients with NSCLC harboring *KIF5B-MET* fusion.

No. Ref	Age	Sex	Smoking status	Stage	KIF5B-MET variant	Histology	Treatment	OS (months)	Mortality Status
1 (12).	NA	NA	NA	NA	*K24*::*M14*	ADC	NA	NA	NA
2 (2).	51	F	never	IV	*K24*::*M14*	ADC	Bvz/Pem-Cis and Pem (PD after 26 mo), SAIT301^#^ (PD), crizotinib (10 mo), then other salvage therapies	>36	Dead
3 (5).	33	F	yes	IV	*K24*::*M15*	ADC	Crizotinib	>8	NA
4 (4).	61	F	never	IV	*K24*::*M15*	ADC+PSC	Pem-Cis	5	Dead
5 (4).	76	M	yes	IV	*K24*::*M15*	PSC	Supportive	1	Dead
6 (6).	46	F	never	IV	*K24*::*M15*	NSCLC	Gem-Cis, crizotinib, nivolumab	7	Dead
7 (3).	56	F	NA		*K24*::*M15*	ADC	CCRT, durvalumab, telisotuzumab, vedotin (8 mo), capmatinib	>34	alive
8 (1).	NA	NA	NA	NA	*K24*::*M15*	ADC	Bvz/Pem-Cis (12 mo), crizotinib (24 mo), cabozantinib (27mo), tepotinib	72	dead

No., number; Ref, reference; ADC, adenocarcinoma; PSC, pulmonary sarcomatoid carcinoma; NSCLC, non-small cell lung cancer; Bvz, bevacizumab; Pem-Cis, pemetrexed-cisplatin; CCRT, concurrent chemoradiotherapy; KIF5B-MET, kinesin family member 5b-hepatocyte growth factor receptor; NA, not available; OS, overall survival; PD, progressive disease; PR, partial response.

## 2 Case description

In May 2020, a 59-year-old woman who had never smoked presented to our hospital with neck pain and limb myasthenia persisting for more than 6 months. Her medical history was unremarkable. However, computed tomography and magnetic resonance imaging revealed a tumor in the lower lobe of her right lung with pleural effusion (**Figure A1**) and multi-organ metastasis, including the brain (**Figure B1**), vertebrae, and bone. Histopathological analysis of tissue biopsy samples obtained from the lower lobe of the right lung using bronchoscopy revealed poorly differentiated adenocarcinoma; the immunohistochemistry results were as follows: napsin A(-), TTF-1(-), CK5/6(-), P40(-), CgA(-), Syn(-), CK7(+), and Ki67(70%+). Next-generation sequencing of the lung biopsy tissue showed *KIF5B-MET* (*K24*::*M15*) fusion without other targeted oncogenic alterations such as *EGFR*, *ALK*, *ROS1*, *BRAF*, *HER2*, *RET*, and *KRAS* (**Table 2**). In addition, the programmed death-ligand 1(PD-L1) expression analysis revealed a tumor proportion score of 40%.

**Figure 1:**
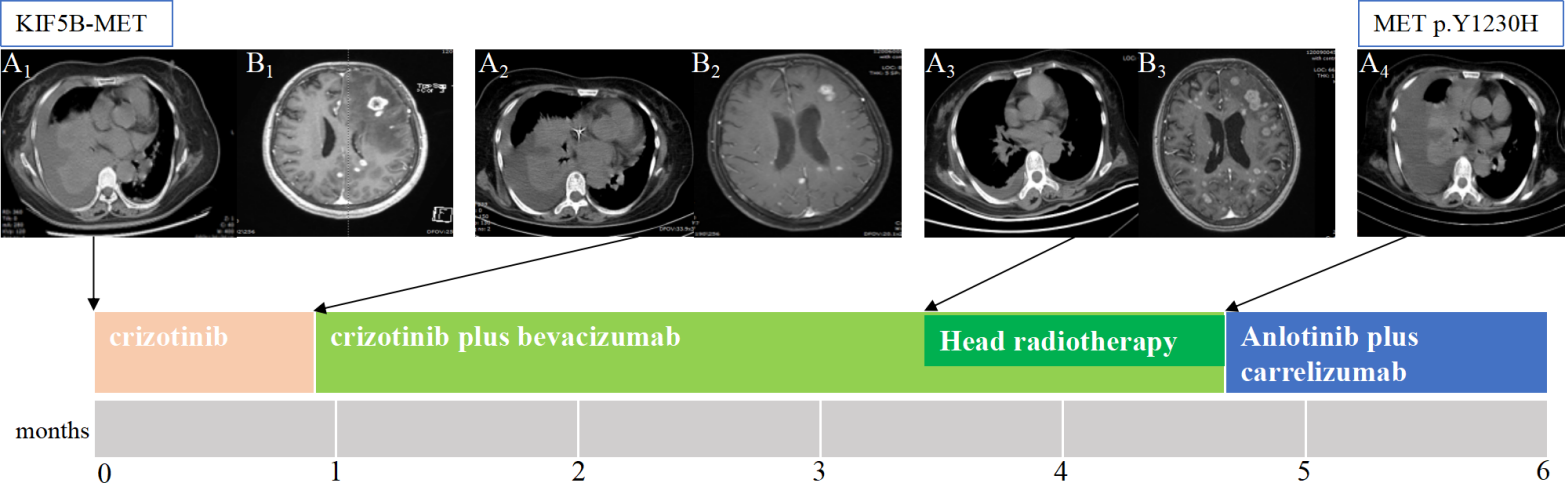
The patient’s clinical course illustrated using lung computed tomography and brain magnetic resonance imaging. **(A1)** Baseline imaging demonstrating abnormal lung mass and pleural effusion in the right lung. **(A2)** After 3 weeks of only crizotinib treatment, the chest CT scan shows that the lung mass and pleural fluid did not change significantly. **(A3)** After crizotinib plus three cycles of bevacizumab, the lung mass shrank, the pleural fluid decreased. **(A4)** After crizotinib plus bevacizumab brain radiotherapy, the chest CT scan shows disease progression, and next-generation sequencing detected the resistance mutation as METp.Y1230H. **(B1)** Brain MRI showing brain metastasis. **(B2)** After 3 weeks of only crizotinib treatment, most of the lesions in the brain slightly decreased, and edema around the lesions decreased, achieving partial response (PR). **(B3)** After crizotinib plus three cycles of bevacizumab, the head lesions grew. CT, computed tomography; MRI, magnetic resonance imaging .

**Table 2:** Molecular alterations of tissue and liquid biopsy.

Timing of test	Type of test	Sample	Result
Diagnosis stage IV	NGS	Tumor	*KIF5B-MET* Fusion *K24*::*M15*
Progression during crizotinib	NGS	Blood	MET NM-000245.3 exon 19 c.3688T>Cp.Y1230H TP53 NM-000546.5 exon 5 c.387-406del

KIF5B-MET, kinesin family member 5b-hepatocyte growth factor receptor; NGS, next-generation sequencing.

## 3 Diagnostic assessment

The patient refused chemotherapy; thus, crizotinib (250 mg, po. bid) was initiated as the first-line treatment in June 2020 without adverse effects.

After 3 weeks of crizotinib treatment alone, a reduction in the size of most lesions in the brain was observed, accompanied by a decrease in edema around the lesions (**Figure B2**). However, the lung mass and pleural fluid did not significantly change (**Figure A2**). Thus, crizotinib was continued owing to its clinical benefits, and bevacizumab was initiated in July 2020.

After crizotinib plus three cycles of bevacizumab, the lung mass shrank, and the pleural fluid decreased (**Figure A3**), but the brain lesions grew (**Figure B3**). Therefore, brain radiation therapy was started in September 2020 (gross tumor volume: 50 Gy/2.5 Gy/20 F, planning target volume: 36 Gy/1.8 Gy/20 F).

By October 2020, the disease had progressed; compared to that in the previous evaluation, the pleural fluid increased, the previously decreased tumor grew, and new lesions appeared in the liver (**Figure A4**). Targeted sequencing was performed on new blood samples using a panel of 483 cancer-related genes to identify new actionable mutations, revealing a novel point mutation in *MET* exon 19 (c.3688T> C; p. Y1230H) (**Table 2**).

The patient again refused chemotherapy after crizotinib plus bevacizumab failure. The MET Y1230H mutant may be sensitive to the type II MET TKI, cabozantinib; however, cabozantinib has not been listed in China. Therefore, we chose anlotinib [a vascular endothelial growth factor receptor-2 tyrosine kinase inhibitor (VEGFR-2 TKI)] plus carrelizumab [an immune checkpoint inhibitor (ICI)] as salvage therapy, from which the patient did not benefit, and her condition worsened. The patient died of multiple organ dysfunction thereafter, surviving 6 months after the diagnosis.

## 4 Discussion

The *KIF5B-MET* fusion gene was originally reported in one of 513 lung adenocarcinoma (LADC) samples (12). There are two known types of *KIF5B-MET* fusions in LADC – *K24*::*M15* and *K24*::*M14*. We identified eight cases in the literature; six involved *K24*::*M15* and two involved *K24*::*M14* (**Table 1**). Thus, our patient with a gene fusion between exon 24 of *KIF5B* and exon 15 of *MET* is the ninth case to be reported.

The *KIF5B-MET* variant has oncogenic functions in cancer cells (4), but *MET* inhibitors have potential therapeutic effects on tumors expressing the *KIF5B-MET* fusion protein. Cho et al. (2) reported the first documented case of a *KIF5B-MET* (*K24*::*M14*) gene rearrangement in a patient with LADC who responded positively to treatment with crizotinib, a *MET* inhibitor. Plenker et al. (3) also reported positive results after 8 months of crizotinib treatment in a patient with a *KIF5B-MET* (*K24*::*M15*) gene fusion. In our case, the patient refused chemotherapy. Thus, crizotinib was taken orally with positive results after 3 weeks; most brain lesions decreased slightly. However, significant lung lesions or pleural fluid changes did not occur.

A multicenter Phase 3 study (CTONG1509) (13) reported that bevacizumab plus erlotinib provides superior progression-free survival compared to erlotinib alone in Chinese patients. In addition, bevacizumab with alectinib has been proven to be a safe and highly effective first-line therapy (14). Bevacizumab is a monoclonal antibody against VEGF; thus, VEGFR2 inhibition enhances the anti-tumor effect of molecularly targeted drugs in various oncogene-driven NSCLC models by inhibiting tumor angiogenesis and exerting a direct antiproliferative effect on cancer cells (15), which suggests that combination therapy with bevacizumab and molecularly targeted agents is a promising strategy for patients with NSCLC harboring oncogenic driver genes. Given the fact that the efficacy of using crizotinib alone was not very satisfactory, we also tried to administer bevacizumab. After treatment with crizotinib plus three cycles of bevacizumab, the lung lesions shrank and pleural fluid decreased; however, the brain lesions increased, possibly due to heterogeneity in the tumor’s response.

Unfortunately, disease progression occurred 1 month later. Therefore, to identify new actionable mutations, targeted sequencing was performed on fresh blood samples using a panel of 483 cancer-related genes, revealing a novel point mutation in *MET* exon 19 (c.3688T> C; p. Y1230H). MET Y1230H is a drug-resistance mutation in the *MET* activation loop, associated with secondary resistance mechanisms in preclinical studies (16). Clinical studies have shown that this mutation is associated with resistance to *MET* inhibitors (17), and structural analysis indicated that it destabilizes the auto-inhibitory conformation of *MET* and abrogates important aromatic stacking interactions with the inhibitor (18). Schrock et al. (19) reported that the MET Y1230 mutation is an acquired mechanism of crizotinib resistance in NSCLC with *MET* exon 14 skipping. Herein, we describe a similar case of a MET Y1230H mutation acquired after crizotinib treatment in a patient with NSCLC, driven by the *KIF5B-MET* (*K24*::*M15*) gene fusion. This patient developed resistance to crizotinib owing to an acquired MET Y1230H mutation. Based on the mechanism of action, MET TKIs are divided into two groups, type I and type II. As type I and II MET TKIs bind the ATP pocket of MET differently, they have distinct inhibition capacities for MET mutants. Engstrom and others confirmed that the MET Y1230H mutant was resistant to type I MET TKIs, such as capmatinib, lvotinib, and crizotinib, but remained sensitive to type II MET TKIs, glesatinib and cabozantinib (20). The switch from a type I MET TKI to a type II MET TKI, cabozantinib, can be an effective strategy to overcome acquired type I MET TKI resistance in NSCLC (21). Unfortunately, to date, cabozantinib has not been listed in China, and the patient was unable to purchase it in China in 2020.

Anlotinib is a small molecule multi-target TKI that can effectively inhibit kinases such as VEGFR, platelet-derived growth factor receptor (PDGFR), fibroblast growth factor receptor (FGFR), and C-Kit. It has anti-tumor angiogenesis and tumor growth inhibition effects. In May 2018, anlotinib hydrochloride capsules were approved for marketing by China National Medical Products Administration, making anlotinib hydrochloride the first drug in China to be approved for third-line treatment of advanced NSCLC. This drug is suitable for the treatment of patients with locally advanced or metastatic NSCLC who have experienced progression or recurrence after receiving at least two types of systemic chemotherapy in the past. The patient refused chemotherapy, hence we administered anlotinib as a third-line treatment. Meanwhile, an ICI was selected as salvage therapy but failed to yield positive outcomes. This is similar to the finding of previous studies that showed that ICIs are less effective for NSCLC with *EGFR* mutation or *EML4-ALK* fusion (22).

In summary, crizotinib can be effective for patients with *KIF5B-MET* fusion; however, cancer cells can develop resistance. In our patient with NSCLC with *KIF5B-MET* fusion, the MET Y1230H mutation was an acquired resistance mechanism to crizotinib. Preventing or overcoming resistance in these patients requires further exploration. ICIs may be ineffective for NSCLC with *KIF5B-MET* fusion.

## Data availability statement

The original contributions presented in the study are included in the article/supplementary material. Further inquiries can be directed to the corresponding author.

## Ethics statement

Written informed consent was obtained from the individual(s) for the publication of any potentially identifiable images or data included in this article. Written informed consent was obtained from the participant/patient(s) for the publication of this case report.

## Author contributions

SD: Conceptualization, Writing – original draft. WD: Conceptualization, Writing – original draft. YT: Data curation, Writing – review & editing. QX: Data curation, Writing – review & editing. TW: Supervision, Validation, Writing – review & editing.
